# Elder abuse in Europe’s “most elderly” city: an update of the phenomenon based on the cases reported to the Penal Court of Genoa from 2015 to 2019 and literature review

**DOI:** 10.1007/s40520-021-01790-6

**Published:** 2021-01-31

**Authors:** Martina Drommi, Alessandra Ponte, Francesco Ventura, Andrea Molinelli

**Affiliations:** Department of Forensic and Legal Medicine, University of Genova, Via De’ Toni 12, 16132 Genova, Italy

**Keywords:** Elder abuse, Domestic violence, Ageism, Personal injury, Prosecutor’s office, Sentence

## Abstract

**Background:**

Elder abuse is currently a worldwide problem. The literature reports that one elderly person out of six is a potential victim.

**Aims:**

To analyse cases reported to the judicial authorities in the territory of Genoa in the period 2010–2019, to investigate the features of elder abuse, to assess the trend of this phenomenon and to propose preventive strategies.

**Methods:**

We analysed the data on reports of abuse passed by the Court of Genoa in the period 2015–2019 concerning physical and mental maltreatment, abandonment and financial exploitation of elderly subjects. These data were compared with those recorded in the previous 5-year period and in the literature.

**Results:**

In the period 2015–2019, 156 cases of elder abuse were identified (versus 63 in the previous period): 18 cases of domestic violence, 5 cases of abuse of the means of correction, 18 cases of caregiver neglect, 76 cases of physical injury and 39 cases of financial exploitation.

**Discussion:**

Abuse was seen to be perpetrated most frequently in the domestic setting and by the victims' relatives. The main risk factors were female gender and the victim’s dependence on others, the maltreating subject’s mental illness and substance abuse.

**Conclusions:**

We documented a progressive increase in the number of abuses reported to the judicial authority; this reflects greater awareness of the problem. However, our figures remained well below the incidence estimated in the literature. It is necessary to train healthcare personnel to identify and manage cases of suspected abuse, and to provide adequate support in situations at risk.

## Introduction

The phenomenon of elder abuse is a highly topical and significant issue. Indeed, it is estimated that, worldwide, one elderly person out of six is a victim of some type of abuse (about 141 million victims per year) [1].

The WHO defines elder abuse as “a single or repeated act, or the lack of appropriate action, occurring within any relationship where there is an expectation of trust which causes harm or distress to an older person” [2]. The phenomenon is extremely serious in view of its possible impact on the life expectancy of elderly subjects; indeed, it gives rise to situations of mental and social distress and increases rates of mortality, hospitalisation and access to emergency departments [3, 4].

Given that the world's population is steadily ageing, the phenomenon of elder abuse is destined to become increasingly common. Data on World Population Ageing indicate that the number of persons aged more than 65 years will increase from 703 million in 2019 to 1.5 billion in 2050 [5]. Moreover, it is estimated that the number of persons aged over 80 years will increase even more markedly, reaching 426 million in 2050. This latter age-group is at greater risk of frailty, isolation and impaired personal autonomy—factors associated with the risk of abuse [6].

In Italy, according to the National Statistics Institute (ISTAT), by 2019 life expectancy at birth had risen to 81 years for men and 85.3 years for women [7]. With regard to the metropolitan area of Genoa, one of Europe’s “most elderly” cities and the focus of our investigation, elderly subjects (over 65 years of age) account for almost 29% of the total population of 841,180. Given the structure of the population and the high index of dependence (47.3%) of the elderly, the territory under examination is one in which the risk of abuse is high.

With regard to the phenomenon of elder abuse, we can distinguish abuse in domestic/community settings [1] and abuse in institutional settings (care facilities, hospitals) [8].

Concerning abuse in the community setting [1], a recent meta-analysis reported that the phenomenon involved 15.7% of subjects aged over 65 years (11.6% psychological abuse, 6.8% financial abuse, 4.2% abandonment and neglect, 2.6% physical abuse, 0.9% sexual abuse).

With regard to abuse in institutional settings, the European Journal of Public Health [8] reported that almost two out of three (64.2%) staff members had reported some form of maltreatment of elderly patients, mainly physical and psychological. Moreover, a survey conducted among the staff of residences for the elderly in the United States in 1993 revealed that about 40% had perpetrated psychological abuse, while 10% had committed acts of physical violence [9]. By contrast, a 2018 Chinese study of elderly people's residences reported a much lower incidence of the phenomenon: of the 681 elderly subjects recruited in two different facilities, 11.48% claimed to have suffered psychological abuse and 8.24% physical abuse [10].

In addition, a US study involving 2,713 elderly persons estimated the incidence of elder abuse in community settings to be about 8.8%; the risk factors identified were: female gender, advanced age (> 85 years) and the presence of multiple physical pathologies. The type of abuse most frequently cited was psychological (4.8%), followed by financial (2.9%), caregiver neglect (1.1%), physical (0.5%) and sexual abuse (0.1%) [11].

The term “physical abuse” refers to the use of force that may result in injury, pain or permanent damage; these actions include violent behaviours, corporal punishment and the improper administration of pharmaceutical drugs.

Psychological maltreatment consists of causing anxiety and/or distress through the use of verbal or non-verbal language and includes threats, intimidation, molestation, and forced isolation of victims from their loved ones. The prolonged state of anguish that may ensue can result in changes of mood and even in full-blown depression [12].

Elderly people, who are often frail and dependent on others, are also vulnerable to theft, fraud or other types of economic exploitation. Financial abuse is defined as the illegal use of an elderly subject’s funds, goods or property, the unauthorised cashing of cheques and falsification of the person’s signature.

Another aspect of elder abuse is that of neglect. For instance, a caregiver may fail to provide for the elderly person’s primary necessities, in terms not only of hygiene, feeding and therapy but also of psychological and social needs [13]. This may be due to the caregiver’s lack of professional training or to wilful neglect on the part of a caregiver who is only interested in being paid and/or obtaining free accommodation [14].

Risk factors for the maltreatment of elderly persons include: female sex, advanced age, cohabitation with the abuser, social isolation and a poor network of support [15]; dementia and other neurological disorders also facilitate abuse, in that caring for such patients places a burden on the caregiver, both physically and, especially, psychologically.

For what concerns the perpetrators of abuse, they are most often members of the elderly person's own family, who are compelled to act as caregivers; this is mainly the case in community settings. In institutional settings, it is the healthcare workers themselves who act as caregivers, and who are therefore at risk of enacting abusive behaviours [16].

The factors that place caregivers at risk of becoming abusers are substance abuse (alcohol or drugs), psychiatric disorders, physical problems, social isolation, a perception of being overburdened with responsibility, and financial or employment problems that may make the subject dependent on the victim [17–19].

In Italy, the family is the mainstay of the care of elderly persons. Consequently, the phenomenon of “informal caregiving” is common; indeed, the elderly are frequently looked after by family members, who often lack the skills needed to carry out this task. Moreover, the commitment undertaken requires the caregiver to live in the same house as the elderly person and to make considerable sacrifices. Indeed, the burden of assistance is added to the ordinary duties of work and family; this may result in economic and social problems, while the elderly person's continual requests may give rise to stress, anxiety and depression [20, 21].

Although the case-records reveal that elder abuse is a serious problem and that its dimensions are inevitably destined to increase, the phenomenon remains underestimated. Indeed, it has been claimed that only 1 in every 24 cases is reported to the judicial authorities [22]. Although the institutions have devoted greater attention to elder abuse over the last decade (owing to media coverage of residential facilities for the elderly), the phenomenon has been only scantly investigated; this hinders the evaluation of its true magnitude and of the adoption of possible preventive measures [23].

In Italy, there is still no specific law that safeguards elderly persons. Indeed, under Italian law, the elderly subject is equated with any subject who is in a state of need, poverty or abandonment [24]. In the Italian Penal Code, the articles that refer more specifically to violence against elderly persons are art. 570 (Violation of the obligations of family care), art. 571 (Abuse of the means of correction or discipline), art. 572 (Maltreatment of family members and cohabiting persons), art. 582 (Personal injuries), art. 591 (Abandonment of minors or incapable persons) and art. 643 (Circumvention of incapable persons). Owing to the intrinsic fragility of elderly persons, these subjects are vulnerable to the offences that we considered, the consequences of which, in prognostic terms, constitute a serious social and economic problem for society.

## Materials and methods

The aim of our study was to analyse cases of elder abuse covered by the above-mentioned articles of the Penal Code that came to the attention of the judicial authority in the metropolitan area of Genoa between 2010 and 2019, in order to examine the characteristics of this abuse and propose preventive strategies.

The data from the period 2015–2019 were obtained with the help of the Public Prosecutor’s Office. Subsequently, these data were compared both with those recorded in a previous study conducted by the Department of Legal Medicine of the University of Genoa, which analysed reports and sentences registered in the previous 5-year period (2010–2014) [25], and with the data reported in the literature. In this way, we were able to depict the present state of the phenomenon and to trace its evolution over the last 10 years.

Examination of the contents of the sentences handed down enabled us to pick out several features of elder abuse in the territory considered: profiles of both abusers and victims (sex, age, psychological profile and possible pathologies of both), the type of relationship between the two, the settings in which the abuse took place and, finally, the verdicts pronounced (conviction or acquittal).

## Results

The following data emerged from the reports (Table 1).

**Table 1:** Analysis of the reports collected in Genoa during the period 2015–2019

Penal code	2015	2016	2017	2018	2019	Total
Art 570 (violation of family care obligations)	7	9	6	1	2	25
Art 571 (abuse of the means of correction or discipline)	0	1	2	2	2	7
Art 572 (maltreatment of family members and cohabiting persons)	26	27	37	43	32	165
Art 582 (personal injury)	127	95	103	85	94	504
Art 591 (abandonment of minors or incapable persons)	8	5	8	9	8	38
Art 643 (circumvention of incapable persons)	43	56	41	46	54	240
Total	211	193	197	186	192	979

A total of 979 offences were reported in the period 2015–2019, as against 300 in the period 2010–2014.

With regard to the abuse of the means of correction or discipline, 7 reports were found; these involved offences of a strictly physical nature (beating, forced restraint, injury) that left visible signs on the victim's body; if such incidents are promptly reported, evidence of the offence can easily be gathered.

A large portion of the offences in our case-records involved maltreatment in the family setting, which accounted for 165 reports. Art. 572 safeguards the person's physical safety, mental salubrity and dignity, and refers to unlawful conduct that is reiterated over time and enacted with the intention of engendering a state of permanent oppression.

Of the offences considered, those involving personal injuries were the most common (504 reports). The cases examined referred to situations in which there was a considerable difference in age between the victim and the abuser, who exploited the elderly person's frailty.

The abandonment of an incapable elderly person (38 reports) was seen to stem chiefly from disputes among the children of an infirm elderly subject, each of whom may, for various reasons, refuse to take on the responsibility of caring for a parent who is no longer self-sufficient.

Finally, financial exploitation (240 reports) proved to be the second most frequent type of abuse, after physical and psychological maltreatment. Solitude, isolation and the presence of organic diseases that impair the individual's critical faculties and ability to distinguish between the good and bad intentions of others are the main risk factors.

It is very important to underline that there is a great difference between notifications of crimes and trial verdicts; indeed, while in the Italian legal system it is very simple to file a notification against someone, considerable evidence is needed to bring the person to trial. For this reason, there are many crime reports but few of them are judged worthy of prosecution.

Analysis of the judicial sentences enabled us to construct profiles of both abusers and abused elderly persons (sex, age, physical and/or mental disorders) and to identify the causes and settings of the abuses committed. We analysed a total of 4500 sentences, only 156 of which involved the abuse of an elderly person (3.5% of the total, versus 2.1% in the previous 5-year period).

The following figures emerged (Table 2).

**Table 2:** Analysis of the sentences passed in Genoa during the period 2015–2019

Penal code	2015	2016	2017	2018	2019	Total	Conviction	Acquittal
Art 570—violation of family care obligations	0	3	5	6	1	15	7	8
Art 571—abuse of the means of correction…	2	2	0	1	0	5	1	4
Art 572—maltreatment of family members…	4	3	2	6	3	18	13	5
Art 582—personal injury	7	19	22	15	13	76	40	26
Art 591—abandonment of…incapable persons	1	1	0	0	1	3	0	3
Art 643—circumvention of incapable persons	8	3	7	10	11	39	19	20
Total	22	31	36	38	29	156	80	76

For what concerns art. 571 (Abuse of the means of correction or discipline) the 5 cases examined occurred in residential facilities for the elderly, the maltreating subjects being nurses or care workers. The unlawful behaviour was seen to have been enacted during night shifts and involved the imposition of uncomfortable positions for long periods, forced immobilisation, and abuse of the means of constraint. Only in one case, however, was the perpetrator convicted; in the other cases, working conditions were acknowledged to be arduous, with few staff members having to deal with large numbers of patients, and constraint devices being applied for long periods to control bouts of psychomotor agitation or delirium. What emerges here is that lack of time and staff shortages exacerbate care-related stress and reduce the quality of the care provided.

Analysis of the sentences regarding art. 572 (Maltreatment of family members and cohabiting persons) turned up 18 cases of abuse perpetrated in the victim's own home, which was shared by the abuser. In 84% of cases, the perpetrator was a son/daughter of the victim; most perpetrators (63%) were affected by problems of alcoholism, substance abuse or psychiatric disorders; moreover, 18 of the 21 maltreating subjects (86%) were males, and 15 of the 21 (71%) were aged over 40 years.

In the case of informal caregivers, a lack of training and an overload of responsibility may lead to conflict, which may then trigger violent behaviour (damage to property) and physical or verbal aggression (insults, humiliation, death threats). Many authors have identified the following risk factors of abuse: parent–child relationship, male sex of the abuser, age over 40 years, substance abuse, alcoholism, psychiatric disorders, financial dependence on the victim, and forced cohabitation between the victim and the abuser.

Victims' children also proved to be the main offenders in cases involving art 582 (Personal injury), with the home again being the most frequent setting of maltreatment. Moreover, the crime of personal injury is often associated with that of maltreatment in the family (art. 572). Indeed, situations of ongoing daily conflict may explode in physical violence that causes fractures, bruises or lacerations, with the result that treatment in the Emergency Department is required and the incident is reported to the judicial authority.

Three cases of neglect came to light. In these cases, the offender was the victim's caregiver, a subject who is specifically responsible for the care and supervision of the elderly person, and the setting of the maltreatment was the home of an elderly person, who was not self-sufficient. The behaviour took the form of dereliction of duty: long absences from the home, neglect and failure to maintain proper hygiene.

Financial abuse figured in 39 sentences. The victims suffered from mental deficiency (Parkinson's disease, dementia, depression) and had a poor social network. Age proved to be a major risk factor; of the 43 victims, 26 (60%) were over 80 years of age, while 8 (19%) were aged between 75 and 84 years. The oldest old (> 85 years) proved to be at the greatest risk of financial abuse, often being isolated and dependent on others. The offenders did not have a daily relationship with their victims, but gained their trust to defraud them; this occurred in 25 cases involving strangers, 7 cases involving caregivers, and 3 cases involving the staff of residential facilities.

## Discussion and conclusions

Having analysed the results of the present study, and after comparing these with the data from the previous 5-year period [25] and those reported in the literature, we were able to draw several conclusions regarding the trend in the phenomenon of elder abuse over the past decade.

With regard to the sex of the victims of abuse, the literature indicates that women are at greater risk. Similarly, in our study, of the total of 249 elderly subjects who had suffered maltreatment, 134 were women and 115 were men. Moreover, women are at greater risk of reiterated abuse and physical and mental oppression.

Regarding the sex of abusers, there is no complete consensus in the literature. However, our study supported the conviction that a majority of cases of abuse are perpetrated by men. Indeed, on a total of 276 persons charged with abuse, 178 were men and 98 women; maltreatment perpetrated by males tended to be physical and violent (personal injury, abuse in the family), while females were more often involved in financial abuse.

Although physical and psychological abuse was seen to be perpetrated most frequently by a single victims' relative, regarding the extra-family context our study has proved that abuses (especially financial abuse) were mainly perpetrated by a group of two or three people. In fact, acting as real criminal organizations, these strangers were able to gain the confidence of the elderly (pretending to be lawyers, bankers or investors) and to obtain money, cheques, goods or property illegally.

The most common setting of abuse was seen to be the elderly person's own home; about 47% of our cases took place in the domestic environment. This can be explained by the fact that in Italy, unlike the US or Northern European countries (where many of the other studies have been conducted), infirm elderly people tend to be looked after at home, and only exceptionally in residential facilities. In our study, only 5 cases (8%) of abuse took place in institutional settings in the period 2010–2014, and only 14 (9%) in the period 2015–2019. Although estimates of the incidence of abuse in residential facilities in Italy are not yet available, such institutions seem to be safer than the domestic environment, not least on account of the adoption of modern systems of staff surveillance.

For what concerns the trend in the phenomenon of elder abuse over the decade considered, it must first be observed that the total number of cases notified differed markedly between the two 5-year periods; in the period 2015–2019, we recorded 979 reports and 156 sentences that met the criteria for inclusion in our analysis, as against 300 reports and 63 sentences in the previous 5-year period. This increase may have been due to the greater awareness of the problem on the part of the public and the judiciary, resulting in the emergence of cases that, in the past, would not have come to light.

The present study confirmed that the most frequent perpetrators of abuse were the children of the victims, followed by spouses and caregivers; in the previous study, however, caregivers occupied he second position. This finding could suggest that owing to greater public awareness of the phenomenon, the families of elderly persons now exercise greater caution in choosing caregivers.

Comparison of the sentences handed down by the Court of Genoa during the two 5-year periods also yielded interesting findings. The data that emerged from the two studies revealed that convictions had increased; during the second period, 51% of those charged with abuse were convicted, as against 35% in the first period. It can, therefore, be claimed that, during the period 2015–2019, more of the cases reported to the judiciary proved to be genuine cases of elder abuse, and that they were dealt with more strictly.

Given the progressive ageing of the global population, there is a need to increase our knowledge of the characteristics of maltreatment and its related mechanisms, to implement adequate prevention and to ensure prompt identification and effective management of the phenomenon. Doctors and the staff of emergency departments, hospitals and residential facilities play a fundamental role in the screening and identification of cases of abuse [26]; this implies the need to implement screening protocols that are agreed upon by the scientific community.

Although the Comprehensive Geriatric Assessment (CGA) constitutes the "gold standard" for the thorough evaluation of the health and well-being of elderly subjects, it cannot be carried out in a short time (e.g. during visits by family doctors or non-geriatric specialists or in the emergency department) [15]. However, questionnaires like the BASE (Brief Abuse Screen for the Elderly) or the CASE (Caregiver Abuse Screen) can investigate suspected abuse during brief clinical examinations; these questionnaires differ in terms of their compilation time, ease of use and time required for personnel training [27].

The investigation of suspected abuse should include the history of the alleged maltreatment, the elderly person's recent and remote medical history, his/her dependence on others, assessment of the subject's functional capacity, identification of the use of physical or pharmacological means of constraint, psychiatric and cognitive evaluation, complete objective examination, and generic and toxicological laboratory tests [28]. Caution must always be exercised to avoid confusing age-related physiological modifications with signs of abuse; the five main pathological conditions that may cause confusion are skin lesions, anomalous bleeding, fractures, malnutrition and anogenital lesions [29, 30].

Interactions with healthcare personnel offer opportunities to recognise signs of abuse and enable suspected cases to be reported to the judicial authorities. Indeed, in the US, the percentage of accesses to the emergency department due to cases of ascertained elder abuse remains very low compared to the estimated prevalence [31]. This reveals the need to rectify the shortcomings in the identification and reporting of cases of abuse, which could be done by introducing training courses for healthcare personnel and implementing clear and appropriate screening protocols.

In particular, health-care professionals should receive adequate clinical-forensic training in order not only to recognize cases of abuse but also to gather evidence that may be useful in criminal proceedings. If possible, after collecting the victim’s details and story, consent shall be required for inspection, collection and correct storage of biological material for judicial purposes. After a general examination, the lesions on the victim’s body must be located, accurately described and photographed, in order to ensure valuable evidence.

The ultimate objective, in fact, should be to improve knowledge of the phenomenon of elder abuse and to adopt a multidisciplinary approach to its management.

A further useful measure could be to introduce "emergency refuges", facilities where the elderly person can be temporarily assisted until such time as the problem can be properly dealt with. Moreover, systems of psychological and financial support should be made available to families who care for infirm elderly relatives.

With regard to cases of abuse by the staff of residential facilities, the causes appear to lie in the inadequate training of healthcare personnel and work-related stress. This implies that adequate training should be provided, workloads should be reduced and relationships between staff and inmates should be improved.

Elder abuse is an extremely complex and heterogeneous problem. Indeed, global prevention of the phenomenon, prompt identification of cases of maltreatment, the safeguard of victims and recognition of the difficulties facing caregivers are delicate issues that require adequate attention. Despite growing awareness of the phenomenon and the proposals mentioned above, elder abuse remains largely unrecognised and infrequently reported to the authorities.

## Data Availability

This article does not involve clinical studies or contain patient data.
